# Predictive Modeling and Integrated Risk Assessment of Postoperative Mortality and Pneumonia in Traumatic Brain Injury Patients through Clustering and Machine Learning: Retrospective Study

**DOI:** 10.3390/biomedicines11112880

**Published:** 2023-10-24

**Authors:** Jong-Ho Kim, Kyung-Min Chung, Jae-Jun Lee, Hyuk-Jai Choi, Young-Suk Kwon

**Affiliations:** 1 Department of Anesthesiology and Pain Medicine, Chuncheon Sacred Heart Hospital, Hallym University College of Medicine, Chuncheon 24253, Republic of Korea; poik99@hallym.or.kr (J.-H.K.); iloveu59@hallym.or.kr (J.-J.L.); 2 Institute of New Frontier Research Team, Hallym University College of Medicine, Chuncheon 24252, Republic of Korea; 3 Department of Neurosurgery, Chuncheon Sacred Heart Hospital, Hallym University College of Medicine, Chuncheon 24253, Republic of Korea; kyungckm1@hallym.or.kr

**Keywords:** traumatic brain injury, surgery, mortality, pneumonia, machine learning, clustering, Glasgow coma scale, midline shift, time between injury and emergency room

## Abstract

This study harnessed machine learning to forecast postoperative mortality (POM) and postoperative pneumonia (PPN) among surgical traumatic brain injury (TBI) patients. Our analysis centered on the following key variables: Glasgow Coma Scale (GCS), midline brain shift (MSB), and time from injury to emergency room arrival (TIE). Additionally, we introduced innovative clustered variables to enhance predictive accuracy and risk assessment. Exploring data from 617 patients spanning 2012 to 2022, we observed that 22.9% encountered postoperative mortality, while 30.0% faced postoperative pneumonia (PPN). Sensitivity for POM and PPN prediction, before incorporating clustering, was in the ranges of 0.43–0.82 (POM) and 0.54–0.76 (PPN). Following clustering, sensitivity values were 0.47–0.76 (POM) and 0.61–0.77 (PPN). Accuracy was in the ranges of 0.67–0.76 (POM) and 0.70–0.81 (PPN) prior to clustering and 0.42–0.73 (POM) and 0.55–0.73 (PPN) after clustering. Clusters characterized by low GCS, small MSB, and short TIE exhibited a 3.2-fold higher POM risk compared to clusters with high GCS, small MSB, and short TIE. In summary, leveraging clustered variables offers a novel avenue for predicting POM and PPN in TBI patients. Assessing the amalgamated impact of GCS, MSB, and TIE characteristics provides valuable insights for clinical decision making.

## 1. Introduction

Traumatic brain injury (TBI) is a global health concern that exacts a heavy toll on lives and well-being across all age groups. The grim statistics reveal its pervasive impact: approximately 4.48 million people have succumbed to trauma-related incidents, constituting 8% of all global fatalities, with TBI accounting for around 2 million of these tragic deaths [1,2,3]. Of particular concern, about 5% of TBI cases necessitate surgical intervention, often marked by elevated postoperative mortality rates. Furthermore, the duration of ventilator support following surgery has been closely associated with patient outcome [1].

The intricate web of connections between the brain and various bodily organs, including the central nervous system and the gastrointestinal tract, is well documented and commonly referred to as the “brain–gut axis” [4,5]. Recent research has unveiled a new axis—the “lung–brain axis” [6,7]. Within the lungs, the exchange of O_2_ and CO_2_ between atmospheric air and the bloodstream is a vital process. Remarkably, the brain, which is the body’s oxygen powerhouse, orchestrates this exchange, with blood CO_2_ levels playing a pivotal role. However, lung function is intricately linked to cerebrovascular health [8] and can be compromised by conditions such as pneumonia, neurogenic pulmonary edema, and acute dyspnea syndrome. Importantly, these respiratory complications are frequently encountered in patients recovering from brain injuries, often stemming from inhalation or ventilator support, and are among the most prevalent respiratory challenges observed alongside acute respiratory failure [9].

Numerous factors exert a profound influence on the outcomes of TBI cases. Swift transfers to healthcare facilities equipped with neurosurgical expertise have been associated with more favorable prognoses [10]. The severity of the injury itself is a critical determinant of mortality rates [11], with prolonged dependence on mechanical ventilation further complicating matters. Notably, ventilator-associated pneumonia emerges as a significant contributor to mortality among patients with cerebral hemorrhage [12], accentuating the peril of extended ventilator reliance in this population. Additionally, individuals grappling with severe brain injuries face an elevated risk of pulmonary aspiration [13]. Postoperative pneumonia (PPN) ranks as the third most prevalent complication in the realm of surgical procedures [14]. PPN not only prolongs hospital stays by an average of 7 to 9 days but also incurs escalated medical expenditures [15,16,17]. These multifaceted factors collectively contribute to the occurrence and severity of PPN.

Machine learning (ML), a subfield of artificial intelligence (AI), possesses the prowess to analyze copious amounts of patient data, discern intricate patterns, and provide accurate predictions and risk assessments—a capability with far-reaching implications in the medical landscape, including predictive analytics [18,19,20,21,22]. Clustering, an indispensable data mining technique, enables the classification of data groups based on shared characteristics, thereby identifying representative central points within these groups [23,24]. In this study, we harness the potential of machine learning to predict postoperative mortality (POM) and PPN in TBI patients. Key determinants, including the severity of TBI denoted by Glasgow Coma Scale (GCS) scores, midline brain shift, and temporal features such as the time elapsed between injury and emergency room admission, serve as foundational variables. In an innovative twist, these three variables are subjected to clustering, amalgamating them into a single novel variable. This novel variable is then examined to explore its impact on predicting the occurrence of POM and PPN.

## 2. Materials and Methods

### 2.1. Study Design

This retrospective study aimed to predict and analyze the impact of three important factors on postoperative mortality and postoperative pneumonia (PPN) in patients undergoing surgical intervention for TBI: the time from injury to hospital admission, the severity of TBI as measured by GCS score, and midline shift of the brain. Additionally, this study investigated the impact of clustering variables derived from these factors on prediction and outcome. The analysis included patient cohort data from January 2012 to July 2022.

#### Ethical Approval/Informed Consent

Prior to commencement, this study received approval from the Clinical Research Ethics Committee of Chuncheon Sacred Hospital (IRB No. 2022-09-007). All research activities adhered strictly to the ethical principles outlined in the Helsinki Declaration. Given that the clinical data utilized were obtained after the conclusion of patient treatment, informed consent was waived for this study, which involved vulnerable participants, specifically individuals with traumatic brain injuries.

### 2.2. Primary Outcomes

The primary outcomes for this study included postoperative mortality (POM) and postoperative pneumonia (PPN). POM was defined as death occurring prior to discharge following surgery, while PPN was diagnosed based on the presence of pneumonia or pulmonary infiltrations evident in chest X-rays, coupled with fever (>38 °C), abnormal white blood cell counts (<4000/mL or >12,000/mL), or positive blood culture findings.

### 2.3. Data Sources and Extraction

Data were sourced from the clinical data repository of Hallym University Medical Center, encompassing data from five hospitals collected over a decade. This comprehensive database houses an array of medical records, prescription information, laboratory test results, and radiological findings. Its extensive data repository allows for the retrieval and analysis of patient data, considering demographic attributes, prescriptions, diagnostic examinations, and treatment details. In addition to structured data, the clinical data repository also contains unstructured textual information, including patient notes, test results, and medication administration records. Several previous studies have successfully utilized data from this repository [19,25,26,27,28,29,30]. Patient selection for analysis was based on a history of TBI and subsequent surgical treatment, as identified through emergency room (ER) records. A list of keywords associated with trauma is provided in Appendix A.

### 2.4. Participants

This study included patients who had undergone surgical intervention for TBI and met the criteria for prediction and analysis as described above. Patients with preoperative pneumonia or incomplete data were excluded from the analysis.

### 2.5. Primary Predictors and Other Features

The main predictors in this study included three essential variables: the time between injury and emergency room (TIE) admission (days), the Glasgow Coma Scale (GCS) score, and the size of midline brain shift (mm), all derived from evaluations performed in the ER. In addition to the above three, we considered a comprehensive set of 33 features, including patient attributes and pre- and postoperative data, to enable early prediction. These included age; sex; surgery duration; obesity (defined as body mass index ≥ 30 kg/m^2^); alcohol and tobacco use; imaging findings; presence of skull fracture; various types of hemorrhage (intracerebral, subdural, epidural, intraventricular, and subarachnoid); American Society of Anesthesiologists physical status; blood and fluid volumes administered during surgery; urine output during surgery; estimated blood loss during surgery; packed red blood cells transfused during surgery; fresh frozen plasma transfused and platelet count; preoperative blood urea nitrogen, creatinine, albumin, sodium, and potassium levels; total duration of ventilator support; ratio of arterial oxygen partial pressure to inspired oxygen fraction (PO_2_/FiO_2_ < 300); and the nature of the initial surgical procedure (burr hole, craniectomy, craniotomy, cranioplasty or endovascular coiling, cooperative surgery).

### 2.6. Data Preprocessing and Machine Learning

#### 2.6.1. Data Preprocessing

The data were categorized into continuous and categorical variables. Continuous data were standardized by centering on the mean and scaling to unit variance [31]. Notably, this study encountered an imbalanced target variable distribution for both POM and PPN. Cases without POM or PPN were more prevalent than those with such outcomes, a disparity known to impact classification accuracy [32]. To mitigate this imbalance, we applied the synthetic minority oversampling technique (SMOTE) [33], which generates new data for the minority class using the k-nearest neighbors algorithm. Datasets were prepared separately for POM and PPN and subsequently divided into training and test sets in an 8:2 ratio. The distribution of POM and PPN instances was evenly randomized between training and test sets.

#### 2.6.2. Machine Learning

Five distinct machine learning algorithms were employed to predict both POM and PPN. These algorithms encompassed logistic regression, random forest, light gradient boosting machine, multilayer perceptron, and support vector machine [34,35,36,37,38]. Additionally, predictions for POM and PPN were made using the balanced random forest algorithm without SMOTE. A balanced random forest randomly undersamples each bootstrap sample to balance it [39]. The construction of prediction models involved training each algorithm with a designated training dataset. Model performance was evaluated using five metrics: area under the receiver operating characteristic curve (AUROC), recall, precision, F1 score, and accuracy. Bootstrapping (n = 1000) was applied to calculate 95% confidence intervals (CIs). We originally built and evaluated a model that included three main predictors and all other variables. After clustering, we built and evaluated a model that included the clustering variable and all other variables instead of the three main variables.

#### 2.6.3. Feature Importance

The importance of features in predicting both POM and PPN was assessed using mutual information (MI), a widely recognized metric in machine learning and feature selection tasks [40,41,42]. It quantifies the dependency or information shared between two random variables, making it a useful measure for assessing the relevance of a feature with respect to the target variable. In the context of feature importance, mutual information can be used to rank or select features based on their relevance to the target variable. Higher mutual information values indicate stronger relationships between a feature and the target [43].

### 2.7. Clustering

To generate a novel predictor, we adopted the agglomerative (bottom-up) hierarchical clustering technique, utilizing the primary predictors as the foundation for the clustering process. Agglomerative hierarchical clustering employs a bottom-up methodology, whereby clusters encompass sub-clusters that progressively branch into additional sub-clusters, thereby constructing a hierarchical structure. The process commences by assigning individual objects to distinct clusters. Subsequent iterations involve the amalgamation (merging) of the closest pair of clusters based on specific similarity criteria. This iterative procedure persists until all data points are encompassed within a single cluster [44]. We analyzed the distance between clusters using a dendrogram. Machine learning and clustering were performed using Anaconda (Python version 3.7; Anaconda, Austin, TX, USA). Each cluster was assigned a unique identifier (cluster number) for identification purposes. To ensure transparency and interpretability, the cluster number was added as an additional categorical feature for each patient in the dataset.

### 2.8. Statistics

In the realm of descriptive statistics, continuous features were represented by medians alongside interquartile ranges, while categorical features were denoted by numbers accompanied by corresponding percentages. Disparities between groups were expressed as absolute standardized differences. To assess the relationship between clustering and POM as well as PPN, odds ratios and their corresponding 95% confidence intervals were computed through logistic regression. Adjusted odds ratios were obtained using backward elimination. Statistical analyses were conducted using SPSS statistical software (version 26.0; IBM Corp., Armonk, NY, USA). Hypothesis testing was conducted in a two-sided manner, with a Bonferroni correction applied (alpha = 0.05/2 = 0.025).

## 3. Results

**Figure 1:**
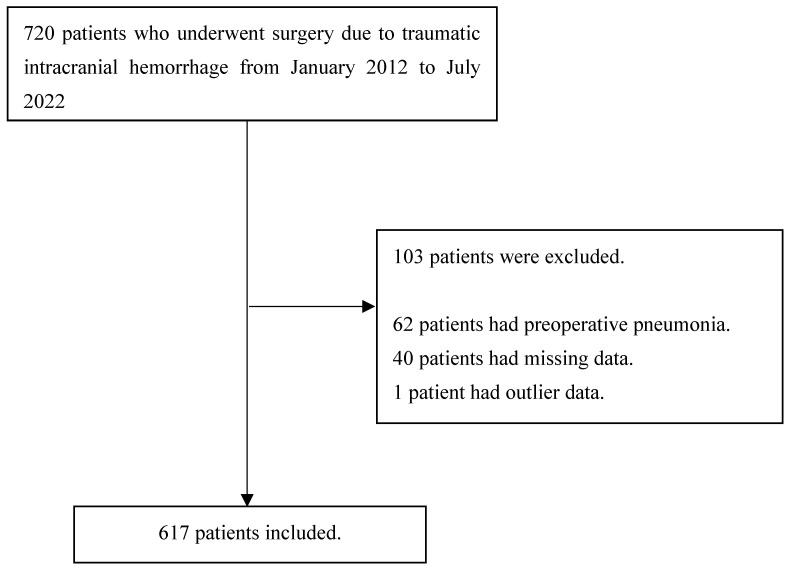
Flow chart.

In the current study, a total of 720 patients were initially considered for inclusion, and 103 patients were subsequently excluded as outlined in Figure 1. Among the remaining 617 patients, 141 (22.9%) unfortunately experienced postoperative mortality, and 185 (30.0%) experienced PPN. Detailed characteristics and perioperative data are comprehensively presented in Table 1. Among the 36 features examined, the ASD for each feature concerning postoperative mortality ranged from 0.0 to 0.8.

### 3.1. Prediction of POM and PPN

The dataset for training comprised 432 patients, while the test dataset included 185 patients. The performance metrics for predicting POM and PPN are summarized in Table 2 and Table 3, respectively.

#### 3.1.1. Performance of POM and PPN Predictions

The first evaluation included a total of 36 features listed in Table 1. For POM prediction, the models achieved AUROC values ranging from 0.63 to 0.72, precision values spanning from 0.38 to 0.47, recall values varying from 0.43 to 0.82, accuracy scores ranging from 0.67 to 0.76, and F1 scores between 0.42 and 0.54. In the case of PPN prediction, the models attained AUROC values within the range of 0.68 to 0.78, precision values from 0.5 to 0.68, recall values spanning 0.54 to 0.76, accuracy scores ranging from 0.7 to 0.81, and F1 scores between 0.55 and 0.69.

#### 3.1.2. Feature Importance

In the context of predicting both POM and PPN, the Glasgow Coma Scale (GCS) exhibited rankings ranging from 7 to 11 for POM and 2 to 25 for PPN. Similarly, the TIE displayed rankings between 10 and 16 for POM and 10 and 18 for PPN. Furthermore, the midline shift of the brain (MSB) revealed rankings spanning from 20 to 33 for POM and 18 to 30 for PPN. For a comprehensive overview of the importance of GCS, TIE, and MSB with respect to the prediction algorithms for POM and PPN, please refer to Table 3. Additionally, detailed information about the importance of other features is provided in Appendix B.

### 3.2. Clustering

**Figure 2:**
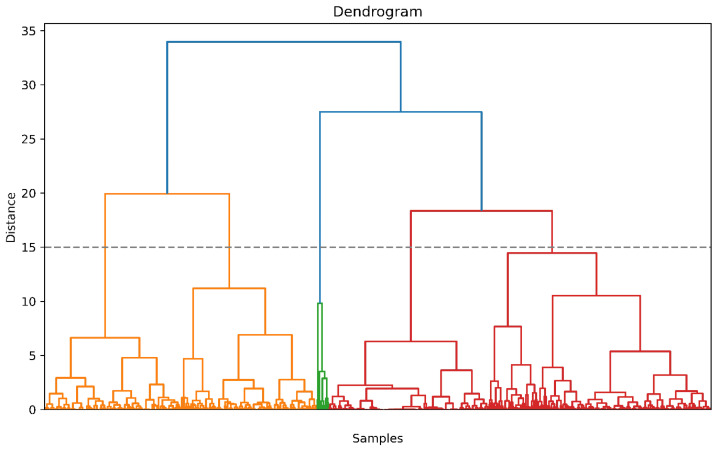
Dendrogram.

**Figure 3:**
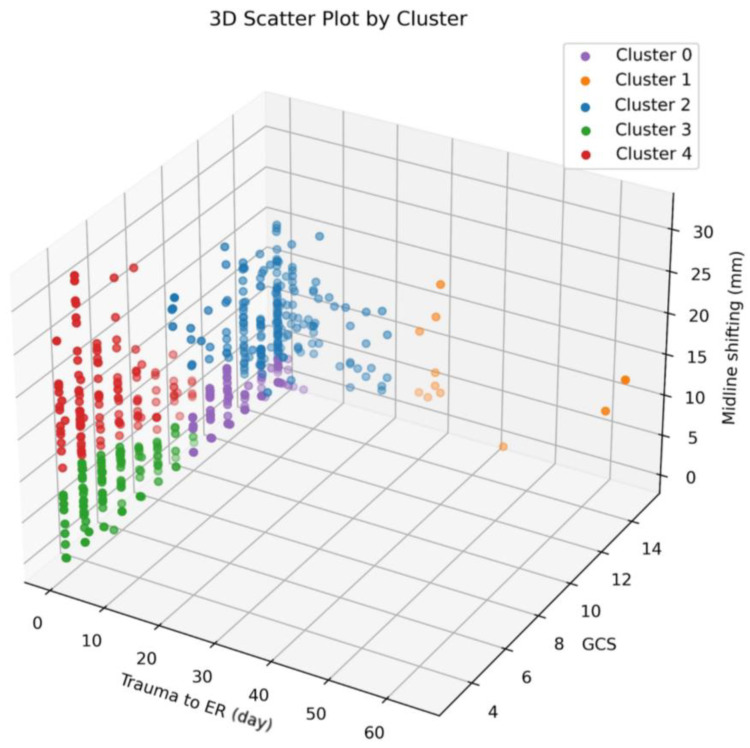
Clustering distribution of patients who underwent surgery due to traumatic brain injury. ER, emergency room; GCS, Glasgow Coma Scale.

The dendrogram, akin to Figure 2, served as a vital tool in determining the optimal number of clusters. Through careful consideration of the number of features employed in clustering, the distances observed between clusters on the dendrogram, and the overall clinical distribution, we determined that the most suitable number of clusters was five. The distribution of patients is depicted graphically in Figure 3.

The number of patients in each of clusters 0 to 4 was 148, 11, 206, 126, and 126, respectively. The number of deaths in each of clusters 0 to 4 was 19 (12.8%), 0 (0%), 20 (9.7%), 49 (38.9%), and 53 (42.1%), respectively. The number of patients experiencing PPN in each of clusters 0 to 4 was 47 (31.8%), 0 (0%), 34 (16.5%), 47 (37.3%), and 57 (45.2%), respectively.

The following is an analysis of the characteristics observed within each cluster:

- Cluster 0 predominantly comprises individuals with high GCS scores ranging from 10 to 15, accompanied by low MSB values spanning from 0 to 7.1 and minimal TIE ranging from 0 to 5.
- Cluster 1 is characterized by elevated GCS scores, ranging from 8 to 15, yet exhibits variability in both MSB (ranging from 0 to 18.1) and TIE (ranging from 27 to 64).
- Cluster 2 generally consists of individuals with high GCS scores, ranging from 8 to 15, paired with low TIE values ranging from 0 to 21. However, the MSB values in this cluster vary between 2.0 and 23.3.
- Cluster 3 is distinguished by low GCS scores spanning from 3 to 10, coupled with low MSB values ranging from 0 to 9.7 and minimal TIE ranging from 0 to 5.
- Cluster 4 features individuals with low GCS scores (ranging from 3 to 10), minimal TIE values (ranging from 0 to 1), and notably high MSB values, which fall within the range of 6.5 to 31.9.

### 3.3. Prediction of POM and PPN with Clustering Feature Added

#### 3.3.1. Performance of POM and PPN Predictions

The model including clustering features was evaluated with 34 features, excluding GCS, TIE, and MSB. The prediction results of POM and PPN with clustering results added as features in addition to the basic features are summarized in Table 4. The models of POM prediction achieved AUROC values ranging from 0.46 to 0.75, precision values ranging from 0.42 to 0.73, recall values ranging from 0.47 to 0.76, accuracy values ranging from 0.42 to 0.73, and F1 scores ranging from 0.33 to 0.67. The models of PPN prediction achieved AUROC values ranging from 0.65 to 0.8, precision values ranging from 0.68 to 0.81, recall values ranging from 0.61 to 0.77, accuracy values ranging from 0.55 to 0.73, and F1 scores ranging from 0.53 to 0.72.

#### 3.3.2. Feature Importance

Within the context of predicting both POM and PPN, the cluster feature exhibited rankings within the range of 5 to 24 for POM and 14 to 37 for PPN. Further details on feature importance, categorized by prediction algorithm for both POM and PPN, can be found in Appendix C.

### 3.4. Odds Ratio of Cluster Variable for POM and PPN

In our analysis, the cluster variable was employed as a categorical factor in logistic regression. Within this framework, we designated cluster 0 as the reference group, distinguished by generally high GCS scores, minimal time elapsed between ER admission and trauma, and limited midline brain shifts. Cluster 3 had a 3.2-fold higher risk of POM than cluster 0. The findings, comprising both unadjusted and adjusted odds ratios for the cluster variable with respect to occurrences of POM and PPN, are succinctly presented in Table 5. 

## 4. Discussion

In this study, we used machine learning to predict postoperative mortality and pneumonia in patients undergoing surgery for TBI. We also investigated the impact of GCS score, MSB, and TIE on prediction. Additionally, we investigated the impact of new variables that cluster these variables on the prediction and risk of POM and PPN. Our analysis included data from a large patient cohort of 617 patients from January 2012 to July 2022. We observed that 22.9% of patients experienced postoperative mortality, while 30.0% experienced postoperative pneumonia. According to the algorithms, the AUCROC for POM and PPN prediction before including the cluster variables was 0.63–0.72 and 0.68–0.78, respectively, and after including the cluster variables, it was 0.46–0.75 and 0.65–0.80, respectively. When calculating the odds ratio according to the new cluster variables, cluster 3 (low GCS score, small midline shift, short time from injury to ER) had a 3.2-fold higher risk of postoperative mortality than cluster 0 (high GCS score, small midline shift, short time from injury to ER).

Abujaber et al., reported the prediction of in-hospital mortality in patients with TBI [45]. They achieved an optimal performance with an accuracy of 95.6% and AUROC of 96%. Midline shift was one of the important features. Hsu et al., predicted the in-hospital mortality rate of TBI patients using GCS score, injury severity scale, and blood pressure, emphasizing the importance of GCS [46]. Lee et al., also predicted the early death of TBI patients using a machine learning model. It is also well known that general anesthesia, surgery, and TBI all affect pneumonia. There have been several studies that have predicted pneumonia in patients who have undergone surgery and TBI. Some studies have used machine learning to predict pneumonia after surgery [47,48,49]. However, there have been no studies on postoperative pneumonia in patients who have undergone surgery due to TBI to our knowledge. There is one reported study on the prediction of pneumonia after TBI. However, previous studies on mortality or pneumonia prediction for TBI included non-surgical patients, so their study population is different from ours.

Our findings follow previous studies highlighting the importance of rapid transfer to a hospital with neurosurgical facilities, high GCS score, and small midline shift of the brain in achieving better outcomes for TBI patients. [50,51,52]. These features have been reported as important factors in the mortality and pneumonia incidence of TBI patients in previous studies using machine learning. GCS score and midline shift were included as important factors [53]. GCS score and MSB may be important factors in hospital-acquired death or early death in TBI patients, and TIE may be an important factor in hospital-acquired pneumonia in TBI patients.

Although we can know the importance of variables in the prediction of machine learning, we cannot know how the variables work in some studies [45,53]. Even if we know how one variable works, we cannot know the effect of integrated information [46]. Clustering variables can have a positive impact on prediction by capturing complex relationships and patterns within data. Depending on the clustering technique used, the resulting clusters themselves can serve as interpretable features [54]. This can facilitate a deeper understanding of how patient subgroups relate to POM and PPN risk. Clustering allowed us to create composite features based on the original predictors. These composite features could represent interactions and combinations of variables that are relevant for predicting postoperative outcomes but may not be explicit in the individual predictors. Clustering can be seen as a form of dimensionality reduction. By grouping similar patients together, we reduced the complexity of the data. This simplification might help the models focus on the most informative aspects of the data, potentially reducing noise. Clustering enabled the stratification of patients into distinct groups based on similarities in their clinical profiles. This grouping might uncover specific subpopulations within the dataset, each with its own risk profile for postoperative outcomes [55]. The inclusion of cluster assignments as categorical features provided a way to interpret model results in the context of these patient subgroups. This can be valuable for clinical decision making. The clustering method can guide doctors in risk assessment, patient stratification, and the development of tailored treatment and monitoring strategies for TBI patients undergoing surgery. We verified the effect of clusters using logistic regression analysis and showed that there were differences in postoperative mortality risk between clusters. This will allow clinicians to assess patients more easily. In a previous study, the time from injury to the emergency room was the most important factor [56]. In our study, we also found that this factor was important in several models, but it was not the most important factor. In our study, we included surgical patients, so anesthesia and surgical factors may have played a role.

This study has the following strengths: First, it provides a foundation for the outcomes by analyzing a significant amount of patient data from 617 patients over a period of 10 years through comprehensive data analysis. Second, it is partially consistent with previous studies that emphasize the importance of factors such as rapid transportation to the emergency room, GCS score, and midline shift of the brain, which has clinical implications for the management of TBI patients. Third, it shows the potential of AI and ML in the medical field by using machine learning algorithms to predict POM and PPN. Fourth, it is innovative to use clustering to generate new variables based on the time from injury to the emergency room and the severity of TBI. This approach allows for a more nuanced understanding of patient subgroups and related risks. However, this study has several limitations. First, this study is retrospective, meaning it relies on past data. This design can introduce bias and limitations related to data quality and the uncontrollability of variables. Second, the study results are based on data from a specific patient population within a specific healthcare system. As healthcare practices and patient demographics can vary, it may be difficult to generalize the results to a wider population. Third, this study uses multiple machine learning algorithms, but the algorithm selection can affect the results. The performance of an ML model can vary depending on the specific algorithm selected. Fourth, external validation of the prediction model on a separate dataset is essential to assess the generalizability and robustness of the results. This study does not mention external validation. Fifth, we did not explicitly determine cutoff values for feature importance, and we used all available features for model development. We are aware of the limitations of the cutoff value determination process. It is important to note that it can be difficult to determine optimal cutoff values for individual algorithms, and there may not be a single solution that applies to all cases. However, feature optimization is needed for performance improvement in future studies.

## 5. Conclusions

This study harnessed the power of machine learning to predict POM and PPN in patients with TBI. Utilizing the GCS score, MSB, and TIE as foundational predictor variables, we embarked on an innovative predictive journey by incorporating clustered variables. These newly derived variables, crafted through advanced clustering techniques, encapsulate critical information from the original predictors, enabling a comprehensive assessment of the amalgamated risk posed by multiple contributing factors within each patient. For future investigations, it is imperative to broaden the scope by encompassing data from diverse healthcare institutions, thus enhancing the generalizability of our clustering-derived variables through rigorous external validation processes. This collaborative effort will advance our understanding of postoperative outcomes in TBI patients and contribute to more effective clinical decision making.

## Figures and Tables

**Table 1:** The patient characteristics and perioperative data.

	Postoperative Survival	Postoperative Death	ASD
Age, years	49 (60–73)	50.5 (61–75)	0.1
Male	345 (72.5)	111 (78.7)	0.1
Duration of surgery, hours	64 (100–150)	79 (110–149)	0.1
TIE	0 (0–4.8)	0 (0–2.4)	0.6
Cooperative surgery	10 (2.1)	1 (0.7)	0.1
Obesity	10 (2.1)	4 (2.8)	0
Alcohol	222 (46.6)	57 (40.4)	0.1
Smoking	149 (31.3)	42 (29.8)	0
Midline shift, mm	2 (5.9–10.5)	3.5 (8.8–15.2)	0.4
Skull fracture	180 (37.8)	60 (42.6)	0.1
Intracerebral hemorrhage	89 (18.7)	25 (17.7)	0
Subdural hemorrhage	317 (66.6)	123 (87.2)	0.5
Epidural hemorrhage	168 (35.3)	23 (16.3)	0.4
Intraventricular hemorrhage	14 (2.9)	13 (9.2)	0.3
Subarachnoid hemorrhage	89 (18.7)	50 (35.5)	0.4
ASA PS	3 (3–4)	3 (3–4)	0.1
Administered blood	0 (200–862.5)	500 (1100–1880)	0.8
Administered fluid	800 (1800–2800)	2100 (3100–4300)	0.8
Urine output	100 (300–600)	175 (360–675)	0.2
Estimated blood loss	200 (600–1500)	1000 (2000–3000)	0.8
Intraoperative PRCs	0 (1–3)	2 (4–6)	0.8
Intraoperative FFP	0 (0–2)	0 (2–3)	0.7
Intraoperative PC	0 (0–0)	0 (0–0)	0.2
Blood urea nitrogen, mg/dL	11.5 (14.5–17.8)	12.5 (14.6–19.7)	0.2
Creatinine, mg/dL	0.7 (0.8–1)	0.7 (0.9–1.1)	0.3
Albumin, g/dL	3.9 (4.2–4.4)	3.8 (4.1–4.4)	0.2
Sodium, mmol/L	137 (139–141)	135 (138–141)	0.1
Potassium, mmol/L	3.4 (3.7–4)	3.1 (3.5–3.8)	0.3
GCS score	8 (13–15)	4 (6–10)	1.1
Total duration of ventilator care	0 (2–10)	3 (6–11)	0.2
O_2_/FiO_2_ < 300	92 (19.3)	67 (47.5)	0.6
Burr hole	103 (21.6)	3 (2.1)	0.7
Craniectomy	155 (32.6)	103 (73)	0.9
Craniotomy	203 (42.6)	28 (19.9)	0.5
Cranioplasty	5 (1.1)	1 (0.7)	0
Coiling	6 (1.3)	5 (3.5)	0.2

ASA PS, American Society of Anesthesiologists physical status; ASD, absolute standardized difference; FFP, fresh frozen plasma; GCS, Glasgow Coma Scale; PC, platelet concentration; PRCs, packed red blood cells; TIE, time between traumatic injury and emergency room.

**Table 2:** AUROC, precision, recall, accuracy, and F1 scores for prediction of postoperative mortality and pneumonia in patients undergoing surgery with traumatic brain injury.

	Model	AUROC (95% CI)	Precision (95% CI)	Recall (95% CI)	Accuracy (95% CI)	F1 Score (95% CI)
POM	Logistic Regression	0.71 (0.61–0.81)	0.46 (0.31–0.63)	0.64 (0.45–0.81)	0.75 (0.68–0.83)	0.54 (0.36–0.67)
	Random Forest	0.63 (0.53–0.73)	0.41 (0.24–0.61)	0.43 (0.25–0.61)	0.73 (0.66–0.81)	0.42 (0.26–0.57)
	Light GBM	0.65 (0.55–0.75)	0.45 (0.26–0.64)	0.46 (0.29–0.65)	0.75 (0.67–0.82)	0.46 (0.29–0.61)
	Multilayer Perceptron	0.69 (0.59–0.79)	0.47 (0.29–0.65)	0.57 (0.39–0.75)	0.76 (0.68–0.83)	0.52 (0.35–0.65)
	SVM	0.64 (0.54–0.74)	0.38 (0.24–0.54)	0.54 (0.36–0.72)	0.7 (0.62–0.78)	0.45 (0.29–0.59)
	Balanced Random Forest	0.72 (0.63–0.81)	0.39 (0.27–0.52)	0.82 (0.68–0.96)	0.67 (0.58–0.74)	0.53 (0.4–0.65)
PPN	Logistic Regression	0.78 (0.7–0.86)	0.68 (0.53–0.83)	0.7 (0.55–0.84)	0.81 (0.74–0.88)	0.69 (0.56–0.8)
	Random Forest	0.74 (0.65–0.82)	0.62 (0.46–0.78)	0.65 (0.5–0.8)	0.77 (0.71–0.85)	0.63 (0.5–0.75)
	Light GBM	0.73 (0.64–0.82)	0.57 (0.42–0.72)	0.68 (0.52–0.82)	0.75 (0.67–0.82)	0.62 (0.48–0.74)
	Multilayer Perceptron	0.69 (0.6–0.78)	0.57 (0.4–0.73)	0.57 (0.41–0.72)	0.74 (0.67–0.81)	0.57 (0.42–0.7)
	SVM	0.68 (0.59–0.77)	0.56 (0.39–0.72)	0.54 (0.37–0.7)	0.73 (0.65–0.81)	0.55 (0.41–0.67)
	Balanced Random Forest	0.72 (0.64–0.8)	0.5 (0.37–0.64)	0.76 (0.61–0.89)	0.7 (0.62–0.77)	0.6 (0.48–0.71)

Prediction of POM and PPN using 36 features. AUROC, area under the receiver operating characteristic curve; CI, confidence interval; GBM, gradient boosting machine; POM, postoperative mortality; PPN, postoperative pneumonia; SVM, support vector machine.

**Table 3:** Feature importance of GCS, TIE, and MSB according to algorithm for prediction of POM and PPN.

	Variable	Logistic Regression	Random Forest	Light GBM	Multilayer Perceptron	SVM	Balanced Random Forest
	GCS	0.201 (10)	0.229 (11)	0.123 (11)	0.204 (7)	0.21 (9)	0.289 (12)
POM	TIE	0.068 (17)	0.182 (16)	0.126 (10)	0.143 (12)	0.162 (13)	0.272 (13)
	MSB	0.131 (13)	0 (33)	0.009 (26)	0.038 (20)	0 (32)	0.1 (24)
	GCS	0.191 (6)	0.275 (9)	0.012 (25)	0.031 (20)	0.07 (17)	0.504 (2)
PPN	TIE	0.049 (22)	0.165 (18)	0.086 (12)	0.103 (10)	0.077 (16)	0.19 (13)
	MSB	0 (27)	0.026 (28)	0.069 (18)	0 (29)	0.002 (27)	0 (30)

Prediction of POM and PPN using 36 features. GBM, gradient boosting machine; GCS, Glasgow Coma Scale; MSB, midline shift of the brain; TIE, time between traumatic injury and emergency room; POM, postoperative mortality; PPN, postoperative pneumonia; SVM, support vector machine.

**Table 4:** AUROC, precision, recall, accuracy, and F1 scores for prediction of postoperative mortality and pneumonia in patients undergoing surgery with traumatic brain injury when a cluster feature is added.

	Model	AUROC (95% CI)	Precision (95% CI)	Recall (95% CI)	Accuracy (95% CI)	F1 Score (95% CI)
POM	Logistic Regression	0.72 (0.62–0.82)	0.64 (0.54–0.75)	0.67 (0.57–0.77)	0.66 (0.55–0.76)	0.6 (0.49–0.7)
	Random Forest	0.46 (0.31–0.62)	0.42 (0.25–0.59)	0.47 (0.29–0.66)	0.42 (0.26–0.58)	0.33 (0.19–0.48)
	Light GBM	0.68 (0.5–0.86)	0.46 (0.29–0.65)	0.5 (0.32–0.68)	0.54 (0.35–0.73)	0.46 (0.28–0.64)
	Multilayer Perceptron	0.75 (0.68–0.82)	0.73 (0.65–0.81)	0.76 (0.68–0.83)	0.73 (0.65–0.8)	0.67 (0.59–0.75)
	SVM	0.55 (0.4–0.68)	0.44 (0.27–0.58)	0.48 (0.3–0.62)	0.47 (0.31–0.61)	0.39 (0.23–0.52)
	Balanced Random Forest	0.72 (0.62–0.82)	0.64 (0.54–0.75)	0.67 (0.57–0.77)	0.66 (0.55–0.76)	0.6 (0.49–0.7)
PPN	Logistic Regression	0.77 (0.69–0.85)	0.77 (0.69–0.86)	0.72 (0.64–0.82)	0.69 (0.6–0.78)	0.67 (0.57–0.76)
	Random Forest	0.65 (0.5–0.8)	0.68 (0.51–0.81)	0.61 (0.46–0.76)	0.55 (0.39–0.7)	0.53 (0.37–0.69)
	Light GBM	0.7 (0.55–0.84)	0.68 (0.53–0.81)	0.62 (0.46–0.78)	0.57 (0.4–0.73)	0.54 (0.37–0.71)
	Multilayer Perceptron	0.8 (0.73–0.86)	0.81 (0.73–0.88)	0.77 (0.69–0.84)	0.73 (0.65–0.81)	0.72 (0.63–0.79)
	SVM	0.68 (0.52–0.79)	0.68 (0.54–0.78)	0.61 (0.46–0.73)	0.56 (0.43–0.68)	0.53 (0.38–0.66)
	Balanced Random Forest	0.77 (0.69–0.85)	0.77 (0.69–0.86)	0.72 (0.64–0.82)	0.69 (0.6–0.78)	0.67 (0.57–0.76)

Prediction of POM and PPN using 34 features. AUROC, area under the receiver operating characteristic curve; CI, confidence interval; GBM, gradient boosting machine; POM, postoperative mortality; PPN, postoperative pneumonia; SVM, support vector machine.

**Table 5:** Odds ratios of the cluster variable for POM and PPN occurrence.

	Postoperative Mortality				Postoperative Pneumonia			
	Unadjusted OR	*p*	Adjusted OR	*p*	Unadjusted OR	*p*	Adjusted OR	*p*
Cluster 0	reference		reference		reference		reference	
Cluster 1	0 (0–0)	>0.999	0 (0–0)	>0.999	0 (0–0)	>0.999	0 (0–0)	>0.999
Cluster 2	0.73 (0.37–1.42)	0.355	0.82 (0.36–1.88)	0.641	0.42 (0.26–0.7)	0.001	0.52 (0.28–0.97)	0.040
Cluster 3	4.32 (2.37–7.87)	<0.001	3.2 (1.54–6.64)	0.002	1.28 (0.78–2.11)	0.336	0.72 (0.39–1.33)	0.290
Cluster 4	4.93 (2.71–8.96)	<0.001	2.32 (1.09–4.92)	0.029	1.78 (1.08–2.91)	0.022	1.07 (0.58–1.97)	0.820

OR, odds ratio.

## Data Availability

Restrictions apply to the availability of these data. Data were obtained from Hallym Medical Center and are available from the clinical data warehouse of Hallym Medical Center with the permission of Hallym Medical Center.

## Appendix A

**Table A1:** Trauma types and the words describing them.

	Word
Slip Down	Slip down
Fall Down	Fall down, Falling, Fall off
Pedestrian Traffic Accidents	Pedestrian, out car
Motorcycle Accidents	Motorcycle
Bump	Collision, Crash, Bump
Bicycle Accidents	Bicycle, Cycle
Car Accidents	In car, Car, Bus, Truck, Driving, Vehicle

## Appendix B

**Table A2:** Feature importance scores according to the algorithm for predicting postoperative mortality and pneumonia.

	Postoperative Mortality						Postoperative Pneumonia					
	LR	RF	LGBM	MLP	SVC	BRF	LR	RF	LGBM	MLP	SVC	BRF
Age	0	0.053	0.086	0	0.113	0	0.149	0.065	0.1	0	0.033	0.059
Male	0	0.09	0.48	0.849	0	0.376	0.258	0.175	0.012	0.39	0.138	0.139
Duration of surgery	0.004	0.09	0.097	0	0.216	0.142	0.086	0.339	0	0.112	0.047	0.26
TIE	0.068	0.182	0.126	0.143	0.162	0.272	0.049	0.165	0.086	0.103	0.077	0.19
Cooperative surgery	0.772	1.006	0.704	0.854	0.107	0.492	0.139	0.378	0.383	0.957	0.244	0.134
Obesity	0	0	0	0.014	0.131	0	0.036	0.063	0	0	0	0.039
Alcohol	0.014	0.084	0.19	0.086	0	0.005	0	0.19	0.046	0	0.008	0
Smoking	0	0.056	0	0	0.124	0.015	0.092	0.02	0	0.017	0	0.072
Midline shift	0.131	0	0.009	0.038	0	0.1	0	0.026	0.069	0	0.002	0
Skull fracture	0.189	0.086	0	0.147	0.155	0.118	0	0.033	0	0	0.045	0.086
Intracerebral hemorrhage	0.061	0.086	0	0.05	0.007	0.122	0	0.07	0	0	0.001	0.092
Subdural hemorrhage	0.613	0.854	0.495	0.709	0.071	0.303	0.197	0.42	0.286	0.43	0.101	0.431
Epidural hemorrhage	0.201	0.209	0.072	0.034	0.163	0.203	0	0	0	0	0.088	0.068
Intraventricular hemorrhage	0	0.122	0	0.025	0.01	0	0.056	0.024	0.082	0	0	0.018
Subarachnoid hemorrhage	0	0.072	0	0.014	0.018	0.005	0	0.132	0.074	0.073	0.083	0
ASA PS	0.308	0.418	0.375	0.342	0.008	0.207	0.109	0.201	0.284	0.313	0.128	0.004
Administered blood	0.137	0.241	0.067	0.11	0.516	0.455	0.108	0.275	0.172	0.033	0.178	0.432
Administered fluid	0.064	0.201	0.031	0	0.128	0.399	0.15	0.217	0.164	0.056	0.188	0.275
Urine output	0	0.077	0.092	0.03	0.222	0.227	0.094	0.183	0.004	0.035	0.093	0.191
Estimated blood loss	0.29	0.286	0.049	0.109	0.329	0.378	0.245	0.222	0.113	0.076	0.134	0.305
Intraoperative PRCs	0.131	0.296	0.1	0.216	0.304	0.566	0.109	0.356	0.12	0.023	0.133	0.462
Intraoperative FFP	0.35	0.406	0.207	0.16	0.116	0.292	0.111	0.261	0.11	0.116	0.299	0.395
Intraoperative PC	0.776	0.618	0.615	0.966	0.174	0.346	0.289	0.279	0.518	0.954	0	0.312
Blood urea nitrogen	0	0.042	0	0	0	0	0.016	0.03	0	0	0.078	0.129
Creatinine	0	0.056	0.017	0	0.034	0	0	0	0	0.01	0.043	0
Albumin	0	0	0	0	0	0	0	0.007	0	0.071	0	0.141
Sodium	0.013	0.19	0	0	0	0.139	0.134	0	0.072	0	0	0
Potassium	0.053	0	0.042	0.035	0.058	0.092	0	0.03	0.015	0	0	0.119
GCS score	0.201	0.229	0.123	0.204	0.21	0.289	0.191	0.275	0.012	0.031	0.07	0.504
Total duration of ventilator care	0.227	0.452	0.297	0.178	0.297	0.486	0.593	0.645	0.55	0.323	0.421	1.021
O_2_/FiO_2_ <300	0	0.019	0.07	0.052	0.108	0.125	0.061	0.107	0.054	0.048	0.121	0
Burr hole	0.125	0.08	0.067	0.019	0.256	0.182	0.102	0.215	0.031	0.021	0.038	0.087
Craniectomy	0.577	0.713	0.235	0.144	0.325	0.464	0.121	0.407	0.076	0.277	0.04	0.215
Craniotomy	0.048	0.228	0.073	0.063	0.209	0.177	0	0.097	0.001	0.102	0.016	0
Cranioplasty	0	0	0	0.02	0	0.005	0.026	0	0.083	0.005	0	0
Coiling	0.08	0.027	0.087	0.138	0.091	0	0	0	0.062	0.034	0.012	0.068

ASA PS, American Society of Anesthesiologists physical status; BRF, balanced random forest; FFP, fresh frozen plasma; GCS, Glasgow Coma Scale; LGBM, light gradient boosting machine; LR, logistic regression; MLP, multilayer perceptron; PC, platelet concentration; RF, random forest; PRCs, packed red blood cells; SVC, support vector machine; TIE, time between traumatic injury and emergency room.

## Appendix C

**Table A3:** Feature importance scores according to the algorithm for predicting postoperative mortality and pneumonia when a cluster feature is added.

	Postoperative Mortality						Postoperative Pneumonia					
	LR	RF	LGBM	MLP	SVC	BRF	LR	RF	LGBM	MLP	SVC	BRF
Age	0	0	0.01	0	0.055	0	0.071	0.003	0	0	0.014	0
Male	0.23	0.298	0.508	0.573	0.259	0.21	0.122	0	0.2	0.443	0.032	0.031
Duration of surgery	0.014	0.116	0.026	0	0.295	0.226	0.076	0.165	0.071	0.101	0.069	0.4
TIE	0.116	0.147	0.058	0.087	0.054	0.229	0.085	0.262	0.129	0.109	0.058	0.191
Cooperative surgery	0.845	0.239	0.564	0.66	0.328	0.421	0.452	0.04	1.033	0.838	0.423	0.146
Obesity	0	0	0	0.003	0	0.004	0.012	0	0	0.075	0.014	0
Alcohol	0.155	0.013	0.056	0	0.163	0.133	0.14	0.01	0.179	0.27	0	0.002
Smoking	0.036	0.004	0.025	0	0	0	0.063	0.022	0.004	0	0	0.159
Midline shift	0.151	0.113	0	0.015	0.059	0.024	0.037	0	0.164	0	0	0.062
Skull fracture	0.3	0.19	0.033	0	0.213	0.226	0.176	0.051	0	0	0.086	0.011
Intracerebral hemorrhage	0.049	0.03	0	0.07	0.083	0.047	0.024	0	0	0	0.064	0
Subdural hemorrhage	1.004	0.487	0.554	0.454	0.412	0.405	0.272	0.148	0.901	0.562	0.455	0.191
Epidural hemorrhage	0.034	0.16	0.231	0	0.069	0.179	0.09	0.035	0	0.005	0.027	0.013
Intraventricular hemorrhage	0	0.009	0.031	0.048	0	0.098	0.005	0	0	0	0.054	0
Subarachnoid hemorrhage	0	0.094	0	0	0	0.014	0	0.085	0.04	0.127	0.022	0.078
ASA PS	0.203	0.164	0.314	0.275	0.28	0.22	0.297	0.088	0.346	0.248	0.323	0.064
Administered blood	0.13	0.337	0.14	0.09	0.344	0.408	0.264	0.283	0.185	0.017	0.103	0.295
Administered fluid	0.094	0.137	0.07	0.119	0.144	0.32	0.219	0.171	0.08	0.082	0.209	0.276
Urine output	0.064	0.056	0	0	0.16	0.1	0.074	0.161	0.053	0	0.064	0.115
Estimated blood loss	0.127	0.221	0.082	0.135	0.279	0.467	0.147	0.155	0.138	0	0.258	0.234
Intraoperative PRCs	0.133	0.176	0.123	0.156	0.152	0.422	0.218	0.278	0.162	0.023	0.112	0.307
Intraoperative FFP	0.19	0.193	0.247	0.129	0.244	0.543	0.127	0.165	0.191	0	0.355	0.087
Intraoperative PC	0.645	0.254	0.338	0.653	0.43	0.499	0.714	0	0.925	0.86	0.336	0.203
Blood urea nitrogen	0	0	0	0	0	0.07	0.136	0.021	0.018	0.012	0.076	0
Creatinine	0	0.021	0	0.025	0	0	0	0	0	0	0.057	0
Albumin	0	0	0	0.024	0	0.077	0	0.146	0	0	0.019	0.168
Sodium	0.02	0.044	0.139	0.079	0.014	0.004	0.129	0.006	0	0.013	0	0.067
Potassium	0.095	0.015	0	0.018	0.043	0.039	0	0.045	0	0	0	0
GCS score	0.19	0.261	0.109	0.173	0.273	0.315	0.192	0.206	0.07	0.013	0.18	0.172
Total duration of ventilator care	0.226	0.39	0.255	0.162	0.352	0.582	0.63	0.737	0.442	0.289	0.46	0.733
O_2_/FiO_2_ <300	0.16	0.052	0	0	0.036	0.027	0	0.05	0.397	0.121	0.065	0.114
Burr hole	0.098	0.148	0.013	0.055	0.23	0.227	0.129	0.162	0.035	0	0.067	0.161
Craniectomy	0.646	0.798	0.235	0.085	0.291	0.303	0.38	0.138	0.202	0.414	0.259	0.134
Craniotomy	0.034	0.206	0.259	0.053	0.172	0.266	0.09	0.025	0	0	0.083	0.017
Cranioplasty	0.063	0.007	0.091	0	0	0.024	0.064	0	0.048	0	0	0.072
Coiling	0.028	0	0	0	0.017	0.043	0.004	0.03	0	0.001	0.029	0
	0.216	0.156	0.013	0.276	0.336	0.17	0	0.1	0.004	0.017	0.032	0.149

ASA PS, American Society of Anesthesiologists physical status; BRF, balanced random forest; FFP, fresh frozen plasma; GCS, Glasgow Coma Scale; LGBM, light gradient boosting machine; LR, logistic regression; MLP, multilayer perceptron; PC, platelet concentration; RF, random forest; PRCs, packed red blood cells; SVC, support vector machine; TIE, time between traumatic injury and emergency room.
